# The Length of Hydrophobic Chain in Amphiphilic Polypeptides Regulates the Efficiency of Gene Delivery

**DOI:** 10.3390/polym10040379

**Published:** 2018-04-01

**Authors:** Ying Zhang, Zhiping Zhou, Mingsheng Chen

**Affiliations:** 1 School of Material Science & Engineering, Jiangsu University, 301 Xuefu Road, Zhenjiang 212013, China; monicazying@163.com; 2 School of Environmental and Chemical Engineering, Jiangsu University of Science and Technology, 2 Mengxi Road, Zhenjiang 212003, China; 3 Department of Pharmaceutical Sciences, College of Pharmacy, University of Michigan, Ann Arbor, MI 48109, USA

**Keywords:** controlled polymerization, biocompatible polypeptides, gene delivery, transfection efficiency, hydrophobic chain, structure-property-function

## Abstract

The major challenges of non-viral carriers are low transfection efficiency and high toxicity. To overcome this bottleneck, it is very important to investigate the structure-property-function (transfection efficiency) relationships of polycations. Herein, different length hydrophobic poly(l-leucine) chains in amphiphilic polypeptides were precisely synthesized by *α*-amino acid *N*-carboxyanhydrides (NCA) ring-opening polymerization and these biocompatible polypeptides were chosen as a model to further examine the transfection in vitro. These polypeptides were characterized by nuclear magnetic resonance spectroscopy (NMR) and size exclusion chromatography (SEC). Agarose gel electrophoresis (AGE) was employed to validate the ability of DNA condensation and transmission electron microscopy (TEM) was used to observe the assemblies of polyplexes. Cytotoxicity was evaluated in COS-7 cell lines and transfection was performed in normal cell COS-7 and cancer cell Hep G2. The results showed that NCA monomers were prepared and the amphiphilic polypeptides, poly(lysine(CBZ))_50_-block-poly(l-leucine)_10_, poly(l-lysine(CBZ))_50_-block-poly(l-leucine)_15_, and poly(l-lysine(CBZ))_50_-block-poly(l-leucine)_25_, were successfully synthesized with controlled molecular weight and narrow distribution. After deprotection of CBZ, these materials can condense plasmid DNA into 100 nm nanoparticles and the cellular uptake of polyplexes was as fast as 30 min. The transfection data shown these materials had a good transfection efficiency comparing to polyethylenimine (Branched, 25 kDa) while they displayed ignored cytotoxicity. More importantly, we discovered the length of hydrophobic poly(l-leucine) in amphiphilic polypeptides steadily regulates gene delivery efficiency in two kinds of cells ranking poly(l-lysine)_50_-*block*-poly(l-leucine)_25_ > poly(l-lysine)_50_-*block*-poly(l-leucine)_15_ > poly(l-lysine)_50_-*block*-poly(l-leucine)_10_.

## 1. Introduction

Nucleic acids have become one of the most important therapeutics for cancer treatment, such as plasmid DNA (pDNA) [1,2,3,4], small (or short) interfering RNA (siRNA) [5,6,7], microRNA (miRNA), messenger RNA (mRNA), and oligodeoxynucleotides (ODN). Unfortunately, efficiency and safety concerns still are two main problems to stop the application of gene therapy mediated by non-viral carriers [8]. To enhance transfection efficiency, correlations of polycation structure and gene delivery efficiency have been extensively investigated, such as molecular weight [9], charge density [10], hydrophobic segments [11], cell penetrating molecules [12], and targeted delivery [13]. Unfortunately, there are still many barriers to enhance the gene delivery while also reducing the toxicity of these materials. Currently, it is especially important to further investigate the correlation between the structure of polymers and transfection efficiency.

**Figure 1:**
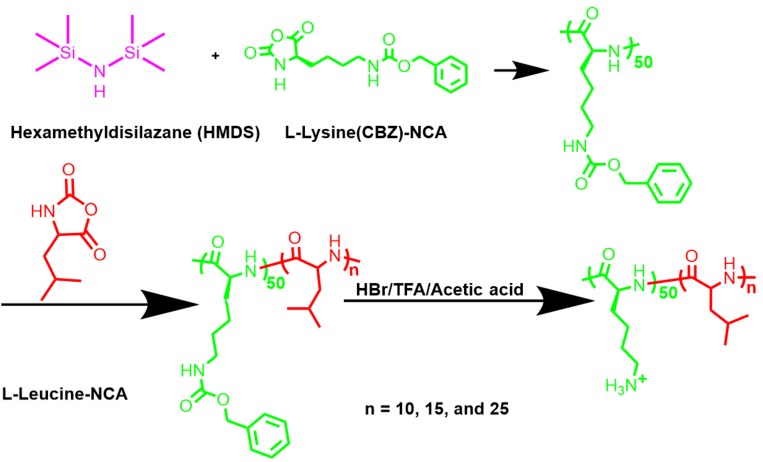
Synthesis route of controlled di-block amphiphilic poly(l-lysine)_50_-block-poly(l-leucine)_n_.

Poly(l-lysine) is a traditional biocompatible material used for gene delivery since the amides can be degradable in the body [14], however, the poly(l-lysine) also has the confliction of cytotoxicity and transfect efficiency, in which high molecular weight poly(l-lysine)s (>30 kDa) possess good efficiency while also having the concern of toxicity, and the low molecular weight poly(l-lysine)s usually behave with low transfect efficiency [15]. Modifying the poly(l-lysine) for better transfection has been widely developed, such as histidylated poly(l-lysine) [16], poly(l-lysine) coating onto functional nanoparticles [17], targeting molecules conjugations [18], and incorporation of hydrophobic chains [19]. Among them, corporation of hydrophobic chains is very attractive to us because it does not increase cytotoxicity. There are many explanations why hydrophobicity can contribute to transfection, while the widely accepted reasons are that the hydrophobic segments can condense the DNA and interact with the hydrophobic lipid layer on the surface of the cells [20]. Poly(l-leucine) is a typical hydrophobic polypeptide and it has been developed for gene or drug delivery [21,22]. Poly(l-leucine) can form an alpha-helical structure [23] and it can interact with phosphatidylglycerol bilayers [24], which gives it the potential to enhance the transfection of low molecular weight poly(l-lysine)s, such as a degree of polymerization (DP) of 50. More importantly, the role of length or number of hydrophobic molecules on transfection efficiency is still unclear. To clarify this issue, we designed controllable biocompatible polypeptides as a model to investigate the efficiency regulation of hydrophobic length in amphiphilic polypeptides (as illustrated in Figure 1) because of the following reasons: First, polypeptides as biocompatible materials, have been widely developed for the delivery of therapeutic agents, including genes, drugs, and vaccines [25,26], and any discovery can contribute its further application. Second, controlled polypeptides can be prepared via *α*-amino acid *N*-carboxyanhydrides (NCAs) ring-opening and it provides us a powerful method to investigate the correlation between the properties of polypeptides and gene transfection efficiency.

In the present work, carboxybenzyl (CBZ)-protected NCAs monomers, l-lysine(CBZ)-NCAs and l-leucine-NCAs, were prepared and then polymerizations were conducted to produce poly(l-lysine(CBZ))_50_-*block*-poly(l-leucine)_10_, poly(l-lysine(CBZ))_50_-*block*-poly(l-leucine)_15_, and poly(l-lysine(CBZ))_50_-*block*-poly(l-leucine)_25_. Following the deprotection of CBZ, amphiphilic polypeptides, poly(l-lysine)_50_-*block*-poly(l-leucine)_10_, poly(l-lysine)_50_-*block*-poly(l-leucine)_15_, and poly(l-lysine)_50_-*block*-poly(l-leucine)_25_ were obtained. The polypeptides were characterized by nuclear magnetic resonance spectroscopy (NMR) and size exclusion chromatography (SEC). Agarose gel electrophoresis (AGE) and transmission electron microscopy (TEM) were employed to validate the ability of DNA condensation. Gene transfection in normal COS-7 cells and cancer Hep G2 cells showed that efficiency was influenced by the length of hydrophobic poly(l-leucine), where the longer hydrophobic block that exists in amphiphilic polypeptides contribute to better transfection efficiency. This research has given a new perspective for the development of gene carriers.

## 2. Materials and Methods

### 2.1. Materials

Triphosgene was bought from TCI (Tokyo, Japan), hexamethyldisilazane (HMDS), H-Lys(Z)-OH (N6-carbobenzyloxy-l-lysine), and l-leucine were purchased from Alfa Aesar (Haverhill, MA, USA). Tetrahydrofuran (THF), dimethylformamide (DMF), and diethyl ether were bought from Sinopharm Chemical Reagent Co. (Shanghai, China), Ltd. 3-(4,5-dimethyl-2-thiazolyl)-2,5-diphenyl-2H-tetrazolium bromide (MTT), Hoechst 33342, hydrogen bromide (33% in acetic acid), chloroquine phosphate, trypsin-EDTA solution 0.25%, branched polyethylenimine (PEI, M_n_ = 10 kDa, M_w_ = 25 kDa, impurities ≤1% water), and rhodamine B isothiocyanate were bought from Sigma-Aldrich (St. Louis, MO, USA). BCA protein kit was purchased from Thermo Fisher Scientific (Waltham, MA, USA). Luciferase assay kit and plasmid DNA (pDNA) pGL3-control vector (5256 bp) for transfection were bought from Promega (Madison, WI, USA). All the cells were obtained from the cell bank of the Chinese Academy of Sciences (Shanghai, China).

### 2.2. Characterization

^1^H NMR spectra was recorded by Bruker nuclear magnetic resonance spectroscopy (400 MHz) with deuterated water (D_2_O) or deuterated dimethyl sulfoxide (DMSO-*d_6_*) as solvents. Molecular weights (MWs) and their polydispersity index (PDI) were determined by size exclusion chromatography (SEC): a Tosoh Corporation SEC (HLC-8320, Tokyo, Japan) was employed to record the polypeptides. Two HLC-8320 columns (TSKgel Super AWM-H, Tosoh Corporation, Tokyo, Japan) and a refractive index detector were equipped. The elution phase was dimethylformamide (DMF) containing 10 mM LiBr with flow rate of 0.6 mL/min, and a series of polymethyl methacrylate (PMMA) was used as calibration standard. Morphologies and size of polyplexes were recorded by a transmission electron microscopy (TEM, JEOL/OXFORD) at a 200 kV accelerating voltage. Polypeptides and plasmid DNA were mixed at N/P = 15 following by 30 min incubation, then a drop of solution was deposited onto 200 mesh carbon film coated copper grids. The grids were placed at 25 °C more than 24 h to evaporate the water. Hydrodynamic size of the polyplexes dispersed in deionized (DI) water was recorded by DLS at 25 °C using a Malvern ZS90 instrument operating at a wavelength of 633 nm and was analyzed by the software of Malvern nanosizer, and the DLS sizes were calculated by number peaks. The additional detailed information: measurement angle is 90°; dispersant RI is 1.330 and the material RI is 1.45; the viscosity (cP) is 0.8872.

### 2.3. Drying of Solvents

Five-hundred milliliters of tetrahydrofuran (THF) and 1.0 g CaH_2_ were added into a clean round-bottom flask and stirred for 24 h. Distillation was conducted in air-free environment to obtain dried THF. The *n*-hexane was dried with the same method as THF. Five-hundred milliliters of DMF and 100 mL toluene were mixed and the trace water in DMF was removed by azeotropic distillation under a N_2_ atmosphere. After cooling to 25 °C, the dried DMF was used for polymerization.

### 2.4. Synthesis of l-Lysine(CBZ)-NCAs Monomer

H-lysine(CBZ)-OH (5.0 g) and triphosgene (5.0 g) were dissolved into dry THF (100 mL) and stirred at 50 °C until the solution become clear. After the solution was concentrated to 50 mL by evaporation, the crude l-lysine(CBZ)-NCAs were precipitated by anhydrous *n*-hexane (250 mL). Then all the l-lysine(CBZ)-NCAs were dissolved in dry THF (50 mL) and then recrystallized by the adding of dry hexane (250 mL). This procedure was repeated 3 times. ^1^H NMR (DMSO, 400 MHz): δ_H_ (ppm) = 9.1 (OCON***H***), 7.24–7.5 (Ar***H***), 5.0 (OCOC***H***_2_), 4.4 (CH_2_CH_2_CH_2_CH_2_C***H***), 3.0 (CONHC***H***_2_). 1.25–1.8 (CONHCH_2_C***H***_2_C***H***_2_C***H***_2_).

### 2.5. Synthesis of l-Leucine-NCAs Monomer

l-leucine (5.0 g) and triphosgene (6.0 g) were added into anhydrous THF (100 mL) and stirred at 50 °C for 6 h at N_2_ atmosphere. l-leucine-NCAs were precipitated by anhydrous *n*-hexane (250 mL) and then added into the mixture of anhydrous THF (50 mL) and *n*-hexane (250 mL) to recrystallize the pure l-leucine-NCAs. The recrystallization was repeated 3 times. ^1^H NMR (DMSO, 400 MHz): δ_H_ (ppm) = 9.1 (CON***H***CH), 4.45 (NHC***H***), 1.75 (CHC***H***_2_), 1.55 (CH_3_C***H***), 0.9 (CHC***H***_3_).

### 2.6. Synthesis of Poly(l-Lysine(CBZ)_50_-Block-Poly(l-Leucine)s

HMDS was used as initiator for the polymerization of NCA monomers. HMDS (100 µL) was dissolved in anhydrous dimethylformamide (DMF), then 50 equivalent l-lysine(CBZ)-NCAs were added quickly. Two days later, 50 µL solution was taken for in situ IR analysis. When the l-lysine(CBZ)-NCAs were consumed (The IR peak at 1790 cm^−1^ disappeared). The original poly(l-lysine(CBZ)_50_ solution was divided into three aliquots quickly (with anhydrous operation). Then three different amounts of l-leucine-NCAs were added to prepare poly(l-lysine(CBZ)_50_-*block*-poly(l-leucine)_10_, poly(l-lysine(CBZ)_50_-*block*-poly(l-leucine)_15_, and poly(l-lysine(CBZ)_50_-*block*-poly(l-leucine)_25_. The reaction solution was monitored by IR and the products were precipitated by ethyl ether after all monomers were consumed. ^1^H NMR (DMSO, 400 MHz): δ_H_ (ppm) = 7.9–8.5 (N***H***CH), 7.0–7.5 (Ar***H***), 5.0 (ArC***H***_2_OCO), 3.8 (COC***H***CH_2_CH), 2.9–3.1 (CONHC***H***_2_CH_2_), 1.0–2.0 (COCHC***H***_2_C***H***_2_C***H***_2_, COCHC***H***_2_C***H***CH_3_), 0.9 (CHC***H***_3_).

### 2.7. Deprotection to Produce Poly(CBZ-l-Lysine)_50_-Block-Poly(l-Leucine)s

One gram of poly(l-lysine(CBZ))_50_-*block*-poly(l-leucine)s was dissolved in trifluoroacetic acid (TFA) and eight-fold hydrogen bromide was added. The solution was stirred for 2 h at room temperature (25 °C). The product of poly(l-lysine)_50_-*block*-poly(l-leucine)s were precipitated by diethyl ether. The polypeptides were vacuumed overnight, or NaOH solution was quickly added to bring the solution to neutral pH and the products were further purified by dialysis against to DI water (MWCO = 3.5 KDa). The result solution was filtrated. ^1^H NMR (D_2_O, 400 MHz): δ_H_ (ppm) = 7.89 (N***H***CH), 4.29 (COC***H***NH), 2.98 (NH_2_C***H***_2_CH_2_), 1.3–2.0 (NH_2_CH_2_C***H***_2_C***H***_2_C***H***_2_, CH_3_C***H***C***H***_2_), 0.9 (CHC***H***_3_). 

### 2.8. Assembly of Poly(l-Lysine)-Block-Poly(l-Leucine) with pDNA

Plasmid DNA (pDNA) and poly(l-lysine)_50_-*block*-poly(l-leucine)s were dissolved in DI water at various N/P ratios and incubated at 25 °C for 30 min. The resulted nanoparticles were imaged by TEM and evaluated by agarose gel electrophoresis (100 V, 60 min).

### 2.9. Investigation of the Biophysical Properties of Polyplexes

For the surface charge evaluation, 1 µg pDNA and poly(l-lysine)50-*block*-poly(l-leucine)s were mixed at indicated N/P ratios and water was added to total volume of 1 mL. The solution was incubated at 25 °C for 30 min and the surface charges of polyplexes were recorded by a Malvern nanosizer, and every test has 3 independent repeats. The stable of polyplexes was investigated by the trypsin degradation experiment. In details, 1 µg pDNA and poly(l-lysine)_50_-*block*-poly(l-leucine)_25_ were mixed at N/P = 15, then water was added to total volume 15 µL. Then 35 µL trypsin (0.25%) was added and incubated at 37 °C. The sample was taken out and then put in a freezer at different time point. Finally, the polyplexes were analyzed by agarose gel electrophoresis (100 V, 60 min).

### 2.10. Toxicity Assessment by MTT

The toxicity of poly(l-lysine)_50_-*block*-poly(l-leucine)s were evaluated by MTT viability assay against COS-7 cells, the residual bromide ion was removed by the polypeptides passing through an anion exchange column as described by Erbacher [27]. In detail, COS-7 cells were subculture every three days and then seeded into a 96-well plate with a density of 8000 cells/well. Twenty-four hours later, the polyplexes solution of poly(l-lysine)_50_-*block*-poly(l-leucine)s and pDNA was prepared with N/P (ratio of cationic nitrogen in polymer to anionic phosphate in DNA) = 5, 10, 15, and 20. Solutions were added into each well. The cells were treated by the polyplexes solution for around 48 h. Thereafter, MTT stock solution (20 µL, 5 µg/µL) was added into every well to lyse the cells. After 4 h incubation, the liquid was removed and 200 µL DMSO was added to dissolve the purple formazan crystals. Finally, the absorbance was determined by a BioTek Elx800 at a wavelength of 490 nm. All the evaluations have three independent repeats.

Cell viability (%) = ([OD_490_]_sample-background_/[OD_490_]_control-background_) × 100%.

### 2.11. Confocal Laser Scanning Microscopy (CLSM)

Rhodamine B isothiocyanate was used for poly(l-lysine)_50_-*block*-poly(l-leucine)_15_ labelling. In details, Rhodamine B isothiocyanate (30 mg) and poly(l-lysine)_50_-*block*-poly(l-leucine)_15_ (1800 mg) (*w*/*w* = 1/60) were dissolved in DI water (50 mL). Then the solution was stirred for 24 h at 25 °C. The unreacted Rhodamine B isothiocyanate was removed by extensive dialysis against to DI water (MWCO = 3.5 KDa, five days). Water was changed four times per day. The product was stored in a freezer for use.

The cellular uptake was investigated by CLSM. In detail, BEL-7402 cells were seeded into a six-well plate with a cell density of 150,000 cells per well, with coverslips were placed above. After 24 h incubation, RB-labeled poly(l-lysine)_50_-*block*-poly(l-leucine)_15_ and plasmid DNA were mixed at N/P = 15 and incubated for 30 min at 25 °C. The polyplexes solution was added at a final RB concentration of 8 µg/mL. The cells were cultured at 37 °C. Thirty minutes later, extracellular nanoparticles were washed by PBS (three times) and the cells were fixed by 4% paraformaldehyde solution. Cells were washed three times using ultra-pure water or neutral PBS. Then Hoechst 33342 was added with a final concentration of 8 μg/mL. Finally, the coverslips were rinsed several times by PBS and imaged with a Leica CLSM.

### 2.12. Gene Transfection

Poly(l-lysine)_50_-*block*-poly(l-leucine)s were dissolved in neutral PBS and sterilized by filtration using a 220 nm Millipore filter. Polyplexes were prepared by mixing poly(l-lysine)_50_-*block*-poly(l-leucine)_10_, poly(l-lysine)_50_-*block*-poly(l-leucine)_15_, and poly(l-lysine)_50_-*block*-poly(l-leucine)_25_, with pDNA (pGL3-control vector) at an optimized N/P ratio. COS-7 and Hep G2 cells were seeded at a density of 10,000 cells/well. After 24 h culture, the medium was replaced by 50 μL fresh serum-free DMEM media. Then, 50 µL polyplexes solution with N/P = 15 was added, a little of 100 µM chloroquine applied to all the polypeptides. Cells were cultured at 37 °C with 5% CO_2_ atmosphere for 4 h. The medium was replaced by fresh with 10% serum. After another two days of incubation, the cells were lysed (Promega lysis reagent and performed according to the protocol) and the expression of the luciferase gene was assessed by luciferase assay kit (Promega). The protein concentration was determined by a BCA protein assay kit. Transfection efficiency was calculated based on relative light units (RLU) of protein expressed by luciferase gene and corresponding protein concentrations. All the evaluations have three independent repeats.

Transfection efficiency = relative luminance intensity (RLU)/protein concentration (mg)

### 2.13. Statistical Analysis

The data was given as the mean ± standard deviation (SD). Statistical significance of differences among the groups was analyzed by using a two-tailed distribution and paired *t*-test. The data was statistically significant when *p* < 0.05.

## 3. Results and Discussion

### 3.1. Synthesis of NCA Monomers

**Figure 2:**
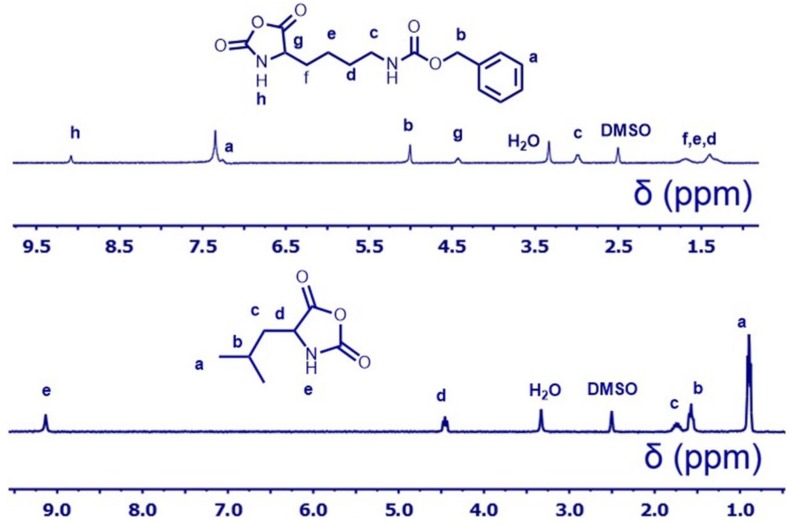
^1^H NMR spectrum of l-lysine(CBZ)-NCA and l-leucine-NCA in DMSO-*d6*.

To investigate the relationship between hydrophobic chain length in amphiphilic polypeptides and the efficiency of gene delivery, controllable polymerization was employed. Until now, several controllable polymerizations have been developed [28,29], such as atom transfer radical polymerization (ATRP), reversible addition-fragmentation chain transfer (RAFT) polymerization, and ionic polymerization. However, the C–C or C–O–C frameworks from ATRP, or RAFT, or ionic polymerization products are too durable and endanger health. Recently, the method for controlled polypeptides were developed using special initiators, such as transition metals and the silicon amine group [30,31]. Concerning the safety issue of residual transition metals, the silane hexamethyldisilazane (HMDS) was chosen as an initiator and the synthesis route was given as Figure 1. For the NCA-opening polymerization, the preparation of pure NCA monomers is the most important. Herein, the l-lysine(CBZ)-NCAs and l-leucine-NCAs monomers were prepared by using triphosgene as the coupling regent. The monomers of l-lysine(CBZ)-NCAs and l-leucine-NCAs were characterized by NMR and the chemical shift was assigned as shown in Figure 2. These results validate the synthesis of pure monomer crystals.

### 3.2. Controllable Polymerization

**Figure 3:**
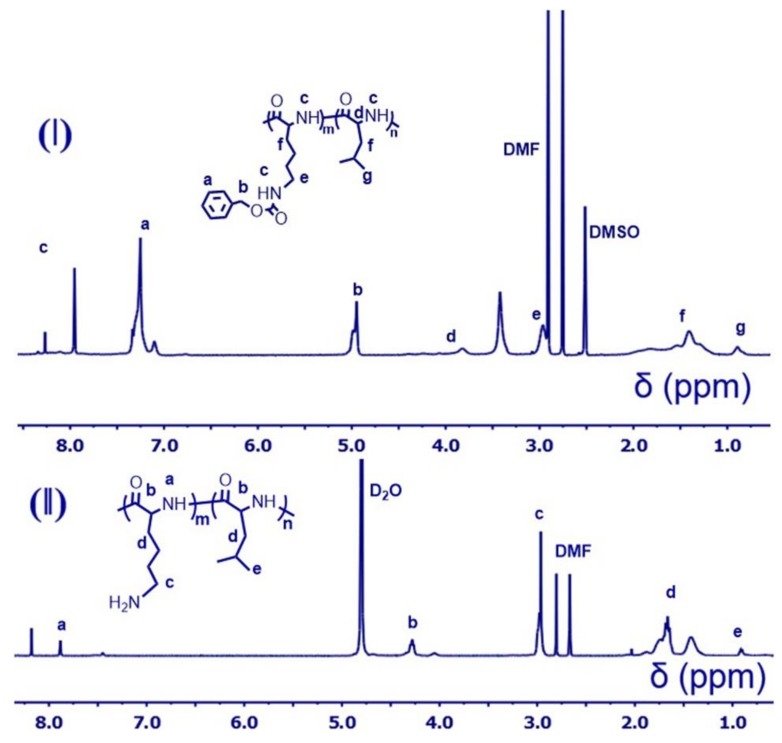
^1^H NMR spectrum of (**I**): poly(l-lysine)_50_-*block*-poly(l-leucine)_10_ in DMSO-d*6* and (**II**): poly(l-lysine(CBZ))_50_-block-poly(l-leucine)_10_ in D_2_O.

The DP of poly(l-lysine(CBZ)) was designed to be 50, and the molecular weight (MW) was determined by size exclusion chromatography (SEC). As shown in Table 1, the MW of poly(l-lysine(CBZ))_50_ (13.2 × 10^3^ g/mol) agreed with the designed value (13.1 × 10^3^ g/mol). From the first block, different amounts of l-leucine-NCAs were added separately to synthesize poly(l-lysine(CBZ))_50_-*block*-poly(l-leucine)_10_, poly(l-lysine(CBZ))_50_-*block*-poly(l-leucine)_15_, and poly(l-lysine(CBZ))_50_-*block*-poly(l-leucine)_25_ with estimated MWs of 14.2 × 10^3^ g/mol, 14.7 × 10^3^ g/mol, 15.9 × 10^3^ g/mol. The SEC-recorded MWs were 14.4 × 10^3^ g/mol, 14.8×10^3^ g/mol, and 15.8 × 10^3^ g/mol correspondingly with a narrow distribution (Table 1), agreeing with the estimated value. The controlled length of the three amphiphilic polypeptides paved a solid foundation for the further research of structure-property-function relationships for these polypeptides. Additionally, the NMR data further certified the polymerization and the peaks of chemical shift were assigned as shown in Figure 3I, validating the successful polymerization. Figures S7–S9 showed the results of different ratio of poly(l-leucine) in the amphiphilic polypeptides, which agreed with the data in Table 1 determined by SEC.

### 3.3. Deprotection to Produce Polypeptides

To dissolve polypeptides in the water phase, the CBZ groups in the poly(l-lysine(CBZ)) need to be eliminated. Therefore, the CBZ groups were removed by HBr, in which the CBZ-protected polypeptides dissolved in TFA first, and then 33% HBr in acetic acid was added to remove the benzyl groups. It needs to be pointed out that this reaction is water sensitive and the CBZ group cannot be removed if the solution was contaminated with trace water [32]. As shown in Figure 3 and Figures S10–S12, the ArCH_2_C*H*_2_ peak at 5.0 ppm and benzyl peaks located at 7–7.5 ppm disappeared after deprotection, validating the elimination of CBZ groups.

### 3.4. Analysis of Biophysical Properties

**Figure 4:**
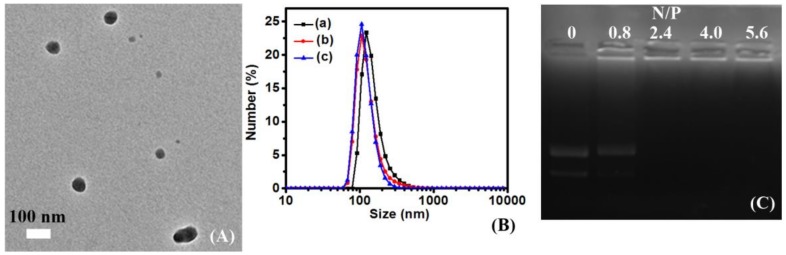
Self-assembling of pDNA and polypeptides: (**A**) transmission electron microscopy of poly(l-lysine)_50_-block-poly(l-leucine)_15_/pDNA at the N/P = 15; (**B**) DLS of (a) poly(l-lysine)_50_-*block*-poly(l-leucine)_25_, (b) poly(l-lysine)_50_-*block*-poly(l-leucine)_15_, and (c) poly(l-lysine)_50_-*block*-poly(l-leucine)_10_ mixed with pDNA at the N/P = 15; and (**C**) agarose gel electrophoresis of poly(l-lysine)_50_-*block*-poly(l-leucine)_15_/pDNA at various N/P ratios.

For DNA therapeutics, there are at least four obstacles to pass for final expression inside cells [33,34]. One of these challenges is to avoid degradation of the exogenous nucleic acids by the host enzyme system. To achieve this goal, cationic polymers, lipids, oligomer, and mesoporous materials were extensively developed [35]. For polycations, the requirement is to condense plasmid DNA into nanoparticles and release DNA at the target site [36]. The importance of DNA condensation potential encourages us to evaluate these polypeptides by agarose gel electrophoresis (AGE) and transmission electron microscopy (TEM). As shown in Figure 4A and Figure S13, all the plasmid DNAs have been condensed into nanoparticles by forming a uniform size around 100 nm at N/P = 15. The AGE data has shown that the DNA can be totally condensed by poly(l-lysine)_50_-*block*-poly(l-leucine)_10_, poly(l-lysine)_50_-*block*-poly(l-leucine)_15_, and poly(l-lysine)_50_-*block*-poly(l-leucine)_25_ at an N/P ratio = 2.4 (Figure 4C and Figure S14). The MW of poly(l-lysine) in amphiphilic polypeptides is around 6.4 KDa (DP = 50) and can still condense the DNA at a low N/P ratio. The DNA condensation property is ideal because the hydrophobic chain can also be serviced for DNA condensation [37,38]. The DLS data shows the sizes of polyplexes were 119 nm, 131 nm, and 154 nm (Figure 4B). The DLS is larger than that observed by TEM and can be explained by the aggregation of polyplexes in aqueous solution [39]. Other scientists think it is the well-known difference between both measurement techniques, as nanoparticles are expanding in solution, while contracting in dry conditions [40].

**Figure 5:**
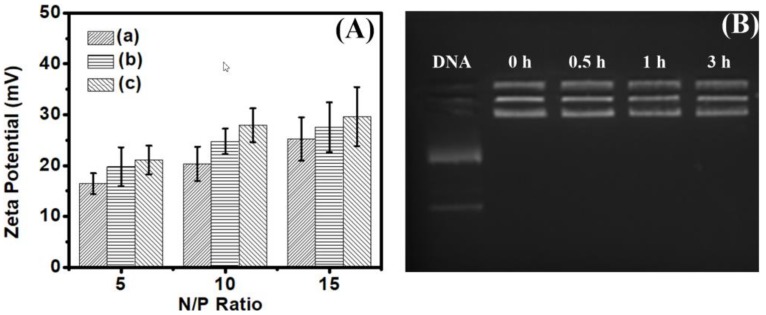
(**A**) Zeta potential of polyplexes of plasmid DNA and (a) poly(l-lysine)_50_-*block*-poly(l-leucine)_10_, (b) poly(l-lysine)_50_-*block*-poly(l-leucine)_15_, (c) poly(l-lysine)_50_-*block*-poly(l-leucine)_25_ at different N/P ratio; and (**B**) polyplexes of poly(l-lysine)_50_-*block*-poly(l-leucine)_25_/DNA digested by trypsin (0.25%) at 37 °C at various time.

We also evaluated the surface charge of these polyplexes. As shown in Figure 5A, when the N/P = 5, the surface charges rank with poly(l-lysine)_50_-*block*-poly(l-leucine)_10_ < poly(l-lysine)_50_-*block*-poly(l-leucine)_15_ < poly(l-lysine)_50_-*block*-poly(l-leucine)_25_. Then, when the N/P rises, the charges become closer. There are the same positive charges in the polyplexes of poly(l-lysine)_50_-*block*-poly(l-leucine)_10_/pDNA, poly(l-lysine)_50_-*block*-poly(l-leucine)_15_/pDNA, and poly(l-lysine)_50_-*block*-poly(l-leucine)_25_/pDNA because the N/P ratio (charge ratio) was used in the preparation of polyplexes. Although the charge density ranks poly(l-lysine)_50_-*block*-poly(l-leucine)_10_ > poly(l-lysine)_50_-*block*-poly(l-leucine)_15_ > poly(l-lysine)_50_-*block*-poly(l-leucine)_25_, the hydrophobic chains also have the function as DNA condensation by the hydrophobic driving force [41], then more primary amines will extend to the surface of the nanoplexes and the surface charge ranks with poly(l-lysine)_50_-block-poly(l-leucine)_10_ < poly(l-lysine)_50_-block-poly(l-leucine)_15_ < poly(l-lysine)_50_-block-poly(l-leucine)_25_. This property, tuned by the hydrophobicity, can possibly regulate the transfection performance.

Traditionally, DNase was used to investigate the stability of polyplexes since the DNA is very sensitive to DNase. The DNA will be degradable quickly in case it releases from polyplexes. We already validate the DNA condensation by agarose gel electrophoresis and TEM and it is very clear that the packed DNA cannot be hydrolytically cleaved by DNase. Herein, we use the trypsin to examine the stability of polyplexes since the trypsin cleaves the chains of polypeptide mainly at the carboxyl side of the amino acids in lysine or arginine. Poly(l-lysine) will be degraded if it releases from the polyplexes. Of course, if the poly(l-lysine) was degraded, the DNA will release out and the agarose gel electrophoresis will detect it. As shown in Figure 5B, after 3 h incubation at 37 °C, the DNA is still protected by amphiphilic polypeptides and seated in the hole of the agar. There is no free DNA out of the hole. This data indicates that the amphiphilic polypeptides can protect the DNA very well, which gives these materials potential further application in gene delivery.

### 3.5. Intracellular Tracking

**Figure 6:**
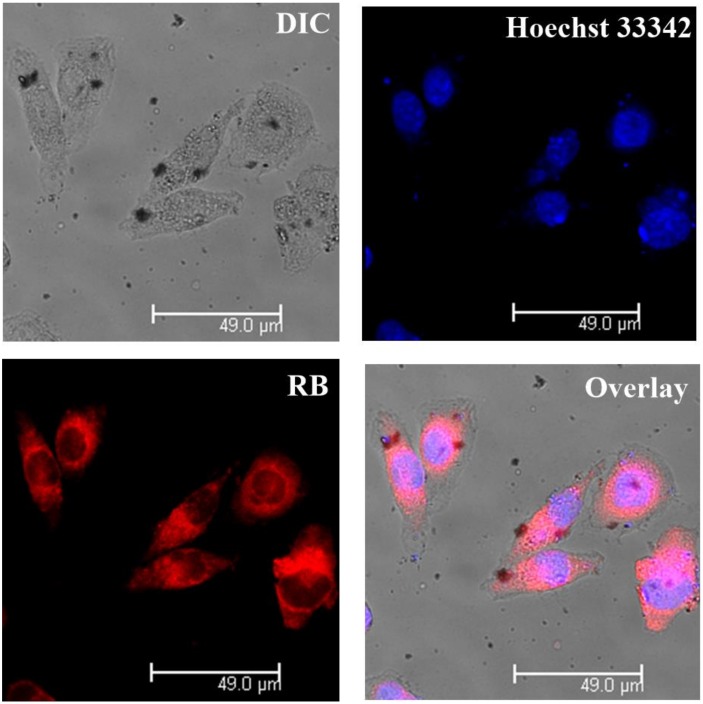
Confocal laser scanning microscopy (CLSM) images of Bel-7402 cells incubated by RB-labelled poly(l-lysine)_50_-*block*-poly(l-leucine)_15_/DNA.

The advantages of nanomedicine not only have enhanced permeability and retention (EPR) effects, but also cellular uptake properties [42,43]. To validate the cellular uptake, confocal laser scanning microscopy (CLSM) images were performed by tracking the RB labeled polyplexes. Due to the similar properties of these three materials, we chose the poly(l-lysine)_50_-*block*-poly(l-leucine)_15_ as a model to record in the BEL-7402 cell line. The poly(l-lysine)_50_-*block*-poly(l-leucine)_15_ was labeled by RB (details were given in the Section 2, Materials and Methods). RB-labeled poly(l-lysine)_50_-*block*-poly(l-leucine)_15_ was mixed with plasmid DNA at N/P = 15 and then the solution was incubated for 30 min. The nanoparticles solution was added into a six-well plate and incubated with BEL-7402 cells for 30 min. The nucleus was stained by diluted Hoechst 33,342 solution. As shown in Figure 6, the nanocomplexes were mainly located in the cytoplasm since the nanoparticles cannot enter nuclei and they are internalized through endocytosis [44,45]. Since the nuclear membrane is a major obstacle for nanoparticles to cross [46], the nanoparticles release the plasmid DNA and then nuclear entry is achieved toward the final protein expression. The present data showed that the amphiphilic polypeptides can deliver the therapeutic DNA into cytoplasm. We also noticed some RB entered the nucleolus, the main reason probably being that the free RB has not been eliminated by dialysis because the amphiphilic polypeptides can form micelles in water. Free RB was wrapped inside micelles and released RB can reach the nucleus. However, it is very clear that the polyplexes can be efficiently taken up by cells and that the polyplexes were mainly restricted within the cytoplasm.

### 3.6. Cytotoxicity Evaluation

**Figure 7:**
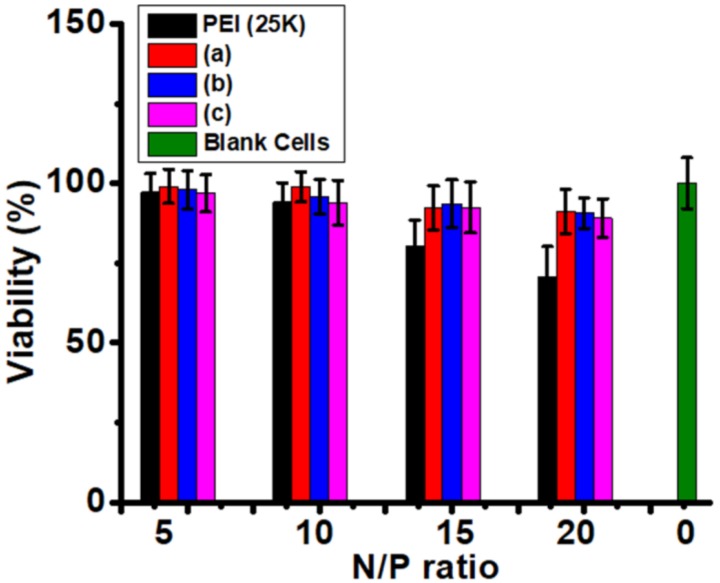
Toxicity assessment of nanocomposites of plasmid DNA and (a) poly(l-lysine)_50_-block-poly(l-leucine)_10_, (b) poly(l-lysine)_50_-block-poly(l-leucine)_15_, (c) poly(l-lysine)_50_-block-poly(l-leucine)_25_, and control (PEI, 25 K) by using COS-7 cells at various N/P (mean ± SD, *n* = 3).

Lowing the cytotoxicity is one of the most important goals for polycations, while most of the high-efficiency gene carriers are highly cytotoxic, such as PEI, PAMAM, and PDMAEMA. Although the poly(l-lysine) is biodegradable material and high MW poly(l-lysine) has little transfection, high cytotoxicity is the fatal weakness for further application. Herein, the MW of poly(l-lysine)_50_ in amphiphilic polypeptides is around 6.4 KDa and these polypeptides should have a relatively low cytotoxicity. As shown in Figure 7, when the N/P rose to 20, many cells treated by PEI were died while the viability of the three polypeptides is higher than 85%. Langer lab reported that the cytotoxicity of poly(l-lysine) is close to PEI (25 KDa) because of the high MW (34 KDa) [15] and low MW poly(l-lysine) in amphiphilic polypeptides reduced cytotoxicity. Additionally, the polypeptides are biodegradable materials since the amide can be hydrolyzed in the body, which means the polypeptides will not be accumulated like durable PEI in the body. This data is the solid evidence for the biocompatible property of these polypeptides.

### 3.7. Gene Transfection

**Figure 8:**
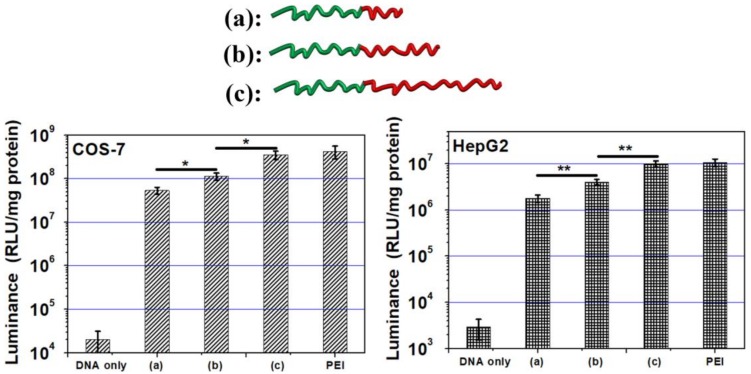
Gene transfection of (**a**) poly(l-lysine)_50_-*block*-poly(l-leucine)_10_, (**b**) poly(l-lysine)_50_-*block*-poly(l-leucine)_15_, (**c**) poly(l-lysine)_50_-*block*-poly(l-leucine)_25_ at their optimized N/P ratio (N/P = 15) in different cell lines (Student’s *t*-test, * *p* < 0.05; ** *p* < 0.01) (mean ± SD, *n* = 3).

High transfection efficiency is a main goal for non-viral gene carriers, however, the traditional material, poly(l-lysine) always displays low transfection. Herein, all the three amphiphilic polypeptides have good transfections in COS-7 and Hep G2 cells. As shown in Figure 8, the transfection of poly(l-lysine)_50_-*block*-poly(l-leucine)_25_ has reached the PEI level and have no obvious difference with PEI (*p* = 0.212 and *p* = 0.276).

As we know, the efficiency of non-viral carriers cannot achieve adequate efficiency as viral vectors. At this moment, the research of structure-property-function as a potential method to overcome all kinds of barrier of non-viral gene carriers triggered our interests. Poly(l-lysine)_50_-*block*-poly(l-leucine)_10_, poly(l-lysine)_50_-*block*-poly(l-leucine)_15_, and poly(l-lysine)_50_-*block*-poly(l-leucine)_25_ were synthesized by controllable NCA-ring opening polymerization. After careful examination, the transfection profiles were displayed in Figure 8, which indicated that the transfection efficiency ranked poly(l-lysine)_50_-block-poly(l-leucine)_25_ > poly(l-lysine)_50_-block-poly(l-leucine)_15_ > poly(l-lysine)_50_-block-poly(l-leucine)_10_ in COS-7 cells (*p* < 0.05) and Hep G2 cells (*p* < 0.01).

Poly(l-lysine) is a typical biodegradable gene carrier, while there are two defects: high cytotoxicity and low efficiency. The main reason of high cytotoxicity is because only high MW poly(l-lysine) (usually >30 K) have a little transfection, while the high MW also resulted in heavy toxicity. Herein, we synthesized small MW Poly(l-lysine) (6.4 KDa). Correspondingly, the materials have an ignored cytotoxicity. The transfection was enhanced by the incorporation of hydrophobic poly(l-leucine), which achieved the PEI (25 KDa) level. More importantly, controlled NCA-ring opening polymerization was employed to produce three kinds of amphiphilic polypeptides, poly(l-lysine)_50_-*block*-poly(l-leucine)_10_, poly(l-lysine)_50_-*block*-poly(l-leucine)_15_, and poly(l-lysine)_50_-*block*-poly(l-leucine)_25_. Interestingly, the transfect efficiency was steadily tuned by the length of hydrophobic poly(l-leucine) in the amphiphilic polypeptides.

We ascribed three main reasons why the hydrophobic chain can enhance gene delivery. First, hydrophobic poly(l-leucine) can interact with the lipid-layer on the surface of cells [47,48], and the longer hydrophobic chain will have a stronger interaction with the cell membrane. Second, the hydrophobic chain can condense DNA via hydrophobic forces [20]. According to the calculation, there will be less N content if there is longer poly(l-leucine) chains in the amphiphilic polypeptides. While the poly(l-lysine)_50_-*block*-poly(l-leucine)_25_ still can condense DNA like poly(l-lysine)_50_-*block*-poly(l-leucine)_10_ (Figure 4 and Figure S14). Third, poly(l-leucine) can form stable *α*-helical conformation when there are 20 residues or more [49]. The longer poly(l-leucine) chain can strength the conformation of *α*-helical and the secondary structure plays an important role in gene delivery [50,51].

## 4. Conclusions

In summary, we have employed the controlled polymerization to tune transfect efficiency by controlling the length of hydrophobic chains in amphiphilic polypeptides, which the transfection ranked poly(l-lysine)_50_-*block*-poly(l-leucine)_25_ > poly(l-lysine)_50_-*block*-poly(l-leucine)_15_ > poly(l-lysine)_50_-*block*-poly(l-leucine)_10_. We noticed some polymeric architectures, such as the degree of branching (DB) in hyperbranched polymers [52], and lipid structure [53], can tune the efficiency of gene delivery. This work will enrich our knowledge of polycation structure-property-transfection efficiency. Additionally, good transfection efficiency and low toxicity (Figure 7) were observed. Usually, poly(l-lysine) has a low transfection efficiency only when the MW is greater than 30 KDa, which also happens to have heavy toxicity [15]. The poly(l-lysine) in amphiphilic polypeptides is 6.4 KDa and, therefore, has a relatively low toxicity (Figure 7), while the transfection efficiency closes to the level of PEI (Figure 8) in two cell lines. The results clearly showed two novelties: First, a new method was developed to promote the transfection efficiency of traditional poly(l-lysine) without obvious high toxicity. Second, we have investigated the structure-property-function relationships of these polypeptides by the regulation of the length of hydrophobic chains in amphiphilic polypeptides and examined the in vitro gene delivery, and found that longer hydrophobic chains exhibit higher gene expression. This discovery illustrates the relationship between the structure of polycations and the transfection potential in gene delivery.

## Figures and Tables

**Table 1:** Results of polymerization.

Entry	Mn. Theory (×10^3^ g/mol)	Mn ^a^ (×10^3^ g/mol)	Mw/Mn	Conv. ^b^	The Final Products	The Proportion of Hydrophobic Domains
Poly(l-lysine(CBZ))_50_	13.1	13.2	1.10	>99%	N/A	N/A
Poly(l-lysine(CBZ))_50_-*block*-Poly(l-leucine)_10_	14.2	14.4	1.14	>99%	Poly(l-lysine)_50_-*block*-Poly(l-leucine)_10_	15.1%
Poly(l-lysine(CBZ))_50_-*block*-Poly(l-leucine)_15_	14.7	14.8	1.27	>99%	Poly(l-lysine)_50_-*block*-Poly(l-leucine)_15_	20.9%
Poly(l-lysine(CBZ))_50_-*block*-Poly(l-leucine)_25_	15.9	15.8	1.3	>99%	Poly(l-lysine)_50_-*block*-Poly(l-leucine)_25_	30.6%

^a^ determined by SEC; ^b^ determined by IR at 1791 cm^−1^.

## Supplementary Materials

The following are available online at http://www.mdpi.com/2073-4360/10/4/379/s1, Figure S1: SEC trace of Poly(l-lysine(CBZ))_50_, Figure S2: SEC trace of poly(l-lysine(CBZ))_50_-*block*-Poly(l-leucine)_10_, Figure S3: SEC trace of poly(l-lysine(CBZ))_50_-block-Poly(l-leucine)_15_, Figure S4: SEC trace of poly(l-lysine(CBZ))_50_-block-Poly(l-leucine)_25_, Figure S5: ^1^H NMR spectrum and integrals of l-lysine(CBZ)-NCA in DMSO, Figure S6:^ 1^H NMR spectrum and integrals of L-leucine-NCA in DMSO, Figure S7: ^1^H NMR spectrum and integrals of poly(l-lysine(CBZ))_50_-*block*-poly(l-leucine)_10_ in DMSO-*d6*, Figure S8: ^1^H NMR spectrum and integrals of poly(l-lysine(CBZ))_50_-*block*-poly(l-leucine)_15_ in DMSO-*d6*, Figure S9: ^1^H NMR spectrum and integrals of poly(l-lysine(CBZ))_50_-*block*-poly(l-leucine)_25_ in DMSO-*d6*, Figure S10:^ 1^H NMR spectrum and integrals poly(l-lysine)_50_-block-poly(l-leucine)_10_ in D_2_O, Figure S11:^ 1^H NMR spectrum and integrals of poly(l-lysine)_50_-*block*-poly(l-leucine)_15_ in D_2_O, Figure S12:^ 1^H NMR spectrum and integrals of poly(l-lysine)_50_-*block*-poly(l-leucine)_25_ in D_2_O, Figure S13: TEM of polyplexes of poly(l-lysine)_50_-*block*-poly(l-leucine)_10_/pDNA (left) and poly(l-lysine)_50_-*block*-poly(l-leucine)_25_/pDNA (right) at N/P = 15, Figure S14: AGE of polyplexes of poly(l-lysine)_50_-*block*-poly(l-leucine)_10_/pDNA (left) and poly(l-lysine)_50_-*block*-poly(l-leucine)_25_/pDNA (right) at various N/P ratios.
