# Supplementary material for: The Length of Hydrophobic Chain in Amphiphilic Polypeptides Regulates the Efficiency of Gene Delivery
Source: Polymers (Basel). 2018 Apr 1;10(4):379. doi: 10.3390/polym10040379 (PMC6415248; doi:10.3390/polym10040379)
Supplement: Supplementary file 1 [file polymers-10-00379-s001.docx]

Supplementary Materials

The Length of Hydrophobic Chain in Amphiphilic Polypeptides Regulates the Efficiency of Gene Delivery

Ying Zhang ^1,2^, Zhiping Zhou ^1,^* and Mingsheng Chen ^3,^*

^1^ School of Material Science & Engineering, Jiangsu University, 301 Xuefu Road, Zhenjiang 212013, China; monicazying@163.com

^2^ School of Environmental and Chemical Engineering, Jiangsu University of Science and Technology, 2 Mengxi Road, Zhenjiang 212003, China

^3^ Department of Pharmaceutical Sciences, College of Pharmacy, University of Michigan, Ann Arbor,
MI 48109, USA

***** Correspondence: zhouzp@ujs.edu.cn (Z.Z.); mingshec@umich.edu (M.C.).


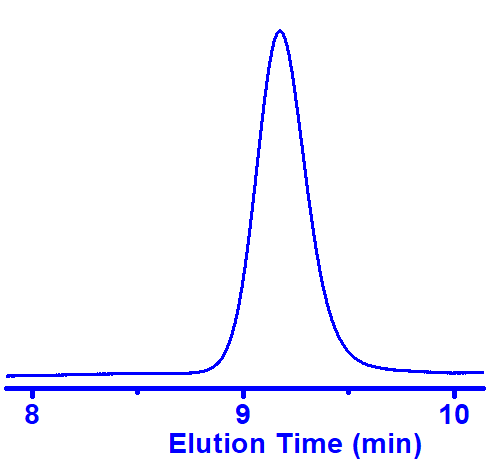


**Figure S1.** SEC trace of Poly(l-lysine(CBZ))*_50_.*


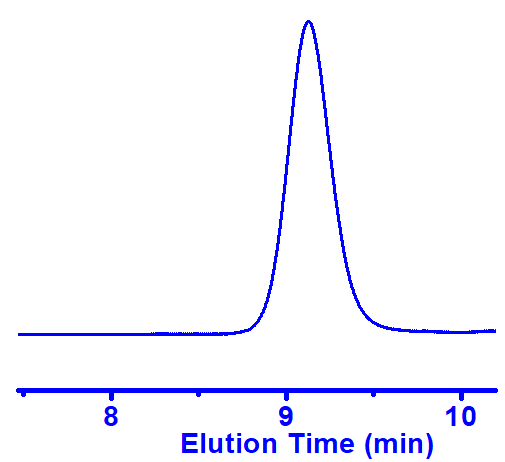


**Figure S2.** SEC trace of poly(l-lysine(CBZ))_50_-*block*-poly(l-leucine)_10._


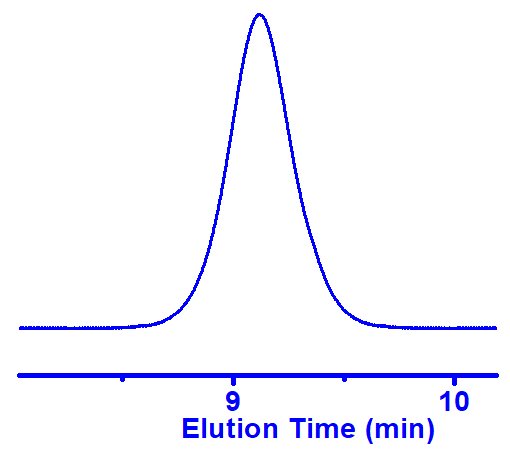


**Figure S3.** SEC trace of poly(l-lysine(CBZ))_50_-block-Poly(l-leucine)_15._


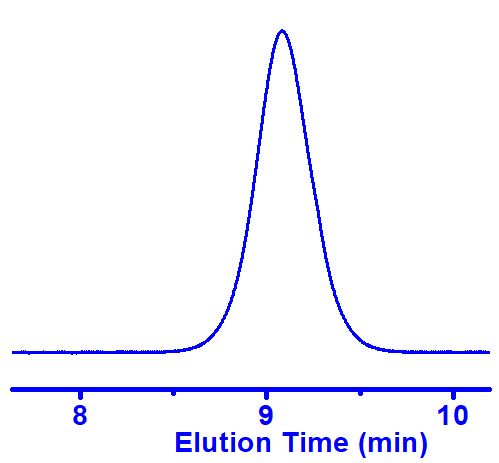


**Figure S4.** SEC trace of poly(l-lysine(CBZ))_50_-block-poly(l-leucine)_25._


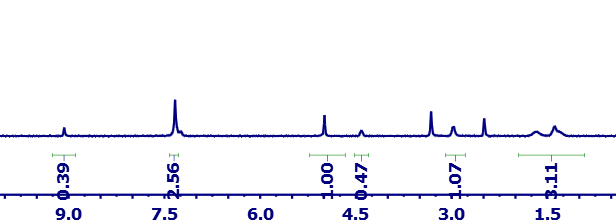


**Figure S5.** ^1^H NMR spectrum and integrals of l-lysine(CBZ)-NCA in DMSO.


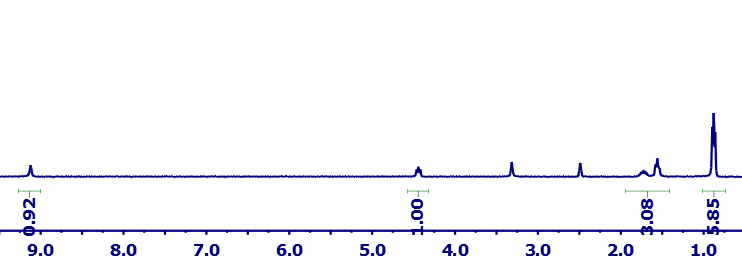


**Figure S6.** ^1^H NMR spectrum and integrals of l-leucine-NCA in DMSO.

**Figure S7.** ^1^H NMR spectrum and integrals of poly(l-lysine(CBZ))_50_-*block*-poly(l-leucine)_10_ in DMSO-*d6*.

**Figure S8.** ^1^H NMR spectrum and integrals of poly(l-lysine(CBZ))_50_-*block*-poly(l-leucine)_15_ in DMSO-*d6*.

**Figure S9.** ^1^H NMR spectrum and integrals of poly(l-lysine(CBZ))_50_-*block*-poly(l-leucine)_25_ in DMSO-*d6*.


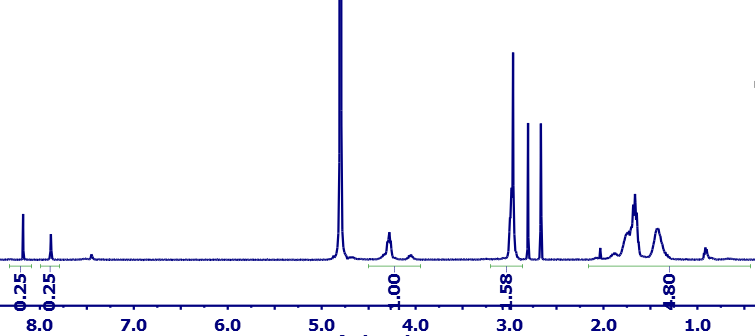


**Figure S10.** ^1^H NMR spectrum and integrals poly(l-lysine)_50_-block-poly(l-leucine)_10_ in D_2_O.


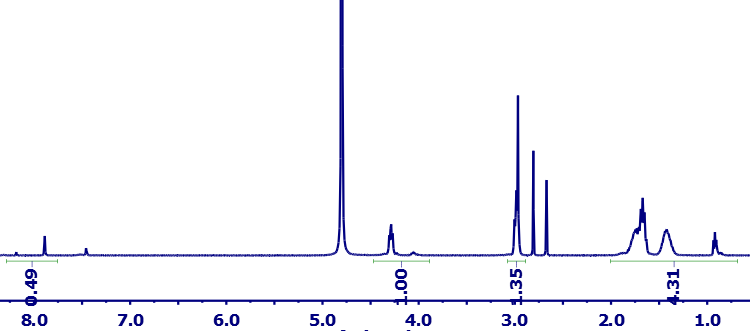


**Figure S11.** ^1^H NMR spectrum and integrals of poly(l-lysine)_50_-*block*-poly(l-leucine)_15_ in D_2_O.


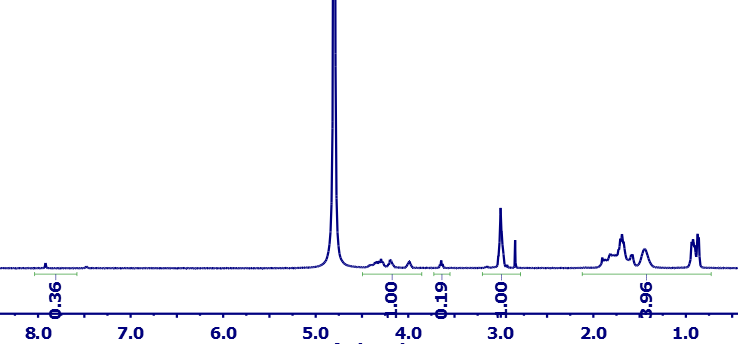


**Figure S12.** ^1^H NMR spectrum and integrals of poly(l-lysine)_50_-*block*-poly(l-leucine)_25_ in D_2_O.


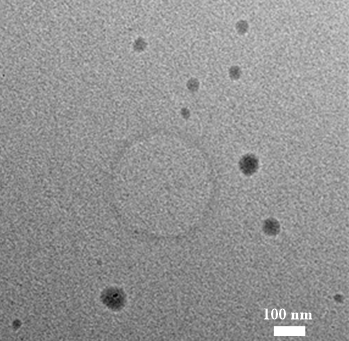

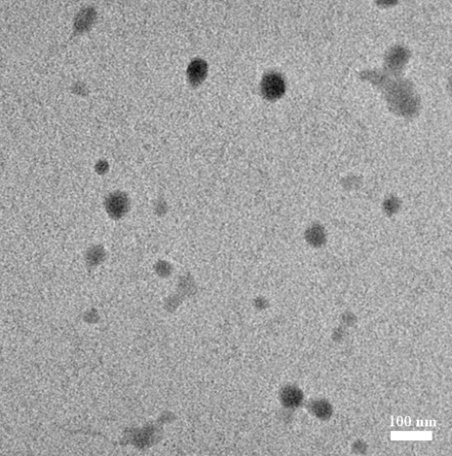


**Figure S13.** TEM of polyplexes of poly(l-lysine)_50_-*block*-poly(l-leucine)_10_/pDNA (left) and poly(l-lysine)_50_-*block*-poly(l-leucine)_25_/pDNA (right) at N/P=15.

**Figure S14.** AGE of polyplexes of poly(l-lysine)_50_-*block*-poly(l-leucine)_10_/pDNA (left) and poly(l-lysine)_50_-*block*-poly(l-leucine)_25_/pDNA (right) at various N/P ratios.

© 2018 by the authors. Submitted for possible open access publication under the
terms and conditions of the Creative Commons Attribution (CC BY) license (http://creativecommons.org/licenses/by/4.0/).
